# Inverted internal limiting membrane flap for the management of optic disc pit maculopathy

**DOI:** 10.5935/0004-2749.20200012

**Published:** 2020

**Authors:** Leandro Cabral Zacharias, Micael Valtoni Dantas do Nascimento, Nagilton Bou Ghosn, Marina Ravagnani Ciongoli, Rony Carlos Preti, Mário Luiz Ribeiro Monteiro

**Affiliations:** 1 Departamento de Oftalmologia, Faculdade de Medicina, Universidade de São Paulo, São Paulo, SP, Brazil

**Keywords:** Tomography, optical coherence, Optic disk, Retinal detachment, Vitrectomy, Case reports, Tomografia de coerência óptica, Disco óptico, Descolamento retiniano, Vitrectomia, Relatos de casos

## Abstract

Optic disc pit is a rare congenital anomaly that can cause serous macular detachment. It has no universally accepted single treatment. Recently, several investigators have performed new procedures to directly seal the pit. Herein, we report a case showing a promising method for optic pit maculopathy surgical treatment. We created an inverted internal limiting membrane flap and fold it over the pit to promote barrier in order to stop further fluid accumulation. Gradual absorption of subretinal fluid was observed over 12 months of follow-up. Optical coherence tomography can demonstrate internal limiting membrane folded over the pit and progressive subretinal fluid resolution. This technique resulted in a satisfactory anatomic outcome with good functional improvement in the best-corrected visual acuity.

## Abstract (pt)

A fosseta do disco óptico é uma rara anomalia congênita que pode causar descolamento de retina seroso na mácula. Não há um tratamento cirúrgico padrão universalmente aceito. Recentemente, cirurgiões têm realizado procedimentos novos que visam selar o buraco diretamente. Esse caso clínico mostra um método promissor para o tratamento cirúrgico da maculopatia causada pela fosseta do disco. Optamos por criar um *flap* invertido com a membrana limitante interna, dobrando-o sobre a fosseta para promover uma barreira, impedindo o acúmulo de fluido. A absorção gradual do líquido subretiniano foi observada ao longo de 12 meses de acompanhamento. Imagens de tomografia de coerência óptica podem demonstrar a membrana limitante interna dobrada sobre a fosseta e a resolução progressiva do fluido subretiniano. Esta técnica resultou em um resultado anatômico satisfatório com boa melhora funcional na acuidade visual.

## INTRODUCTION

Optic disc pit (ODP) is a rare congenital anomaly that causes serous macular detachment (SMD) in 25%-75% of cases. It has no universally accepted single treatment, since no studies have shown a clearer evidence than those in others. This is partly due to the rarity of this clinical condition and partly due to the challenging nature of retinal detachment^(1)^.

A number of treatment options have been explored, ranging from laser photocoagulation, intravitreal gas injection, and macular buckling surgery to pars plana vitrectomy (PPV). Some investigators have performed new procedures to directly seal the pit. The rationale involved is to prevent passage of fluid into the intraretinal and subretinal spaces, using an autologous scleral flap, by injecting autologous platelets, or using a tissue fibrin sealant^(2)^.

Herein, we report a promising method for optic pit surgical treatment, sealing the ODP with inverting peeled internal limiting membrane (ILM).

## CASE REPORT

**Figure 1:**
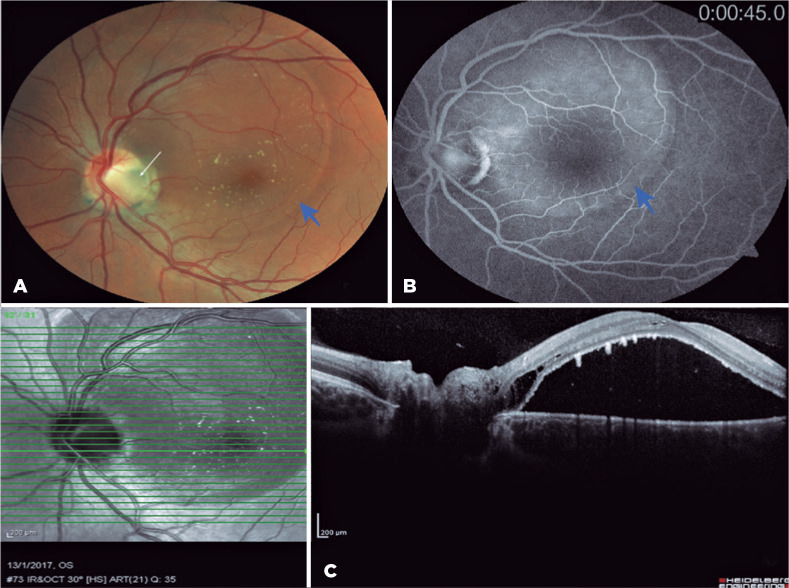
(A) Color fundus image of the left eye at presentation showing a superotemporal optic disc pit (white arrow) with serous macular detachment (blue arrow). (B) Delineated late hyperfluorescence corresponding to the area of macular elevation on fluorescein angiography (blue arrow). (C) Preoperative spectral domain optical coherence tomography shows macular detachment.

A 27-year-old woman presented with visual loss over the last 4 months. On ophthalmologic examination, her best-corrected visual acuity (BCVA) was 20/20 in the right eye (OD) and 20/60 OS. The anterior segment examination was normal in both eyes. Fundus examination OD was unremarkable, whereas OS fundus examination and optical coherence tomography (Spectralis OCT Heidelberg Engineering, Heidelberg, Germany) showed an ODP with SMD (Figure 1).

**Figure 2:**
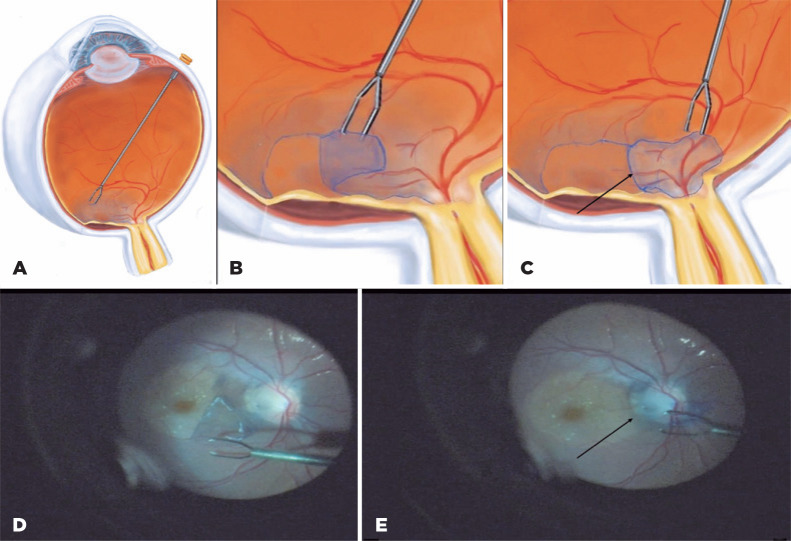
Pars plana vitrectomy with inverted ILM flap confection. Schematic drawings demonstrate (A) the surgeon’s view; (B) the creation of the ILM flap temporal to the fovea and (C) the peeled ILM attached by a pedicle and folded over the optic disc pit. (D) Intraoperative photo. ILM is carefully peeled off from the macular area. The ILM flap should not be torn (E) Intraoperative surgeon’s view. The peeled ILM is inverted and lying over the optic disc. Both black arrows indicate areas where the peeled ILM is left with an attachment acting as a pedicle.

After obtaining a signed informed consent, a 23-gauge vitrectomy was performed. Posterior vitreous detachment was achieved with the help of triamcinolone acetonide. Considering its physiopathology, an inverted ILM flap was created and folded over the pit to promote barrier in order to stop further fluid accumulation, as previously described^(3)^ (Figure 2).

The ILM was stained with brilliant blue G, and a two-disc-diameter flap was carefully created from the temporal to nasal region. The flap was then inverted over the pit and stuffed inside it. A perfluorocarbon liquid drop was used to stabilize the flap during fluid-air exchange, and 10% C3F8 was chosen as intraocular tamponade. The patient was instructed to perform face-down positioning for 5 days.

**Figure 3:**
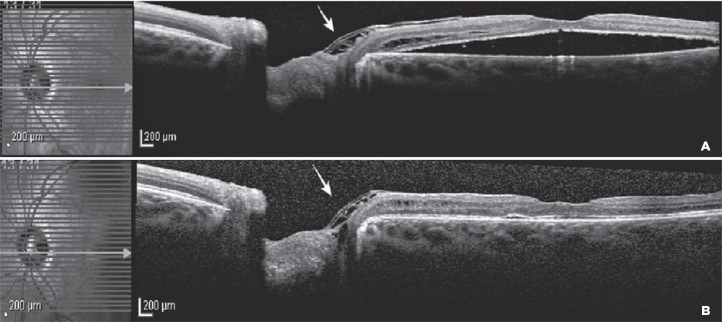
(A) Spectral domain optical coherence tomography (SD-OCT) 3 months postoperatively showing gradual regression of the macular detachment and confirming the presence of ILM covering the optic disc pit (arrow). (B) SD-OCT 12 months postoperatively revealing completely reattached retina with restored foveal contour and ODP overlying the ILM flap (arrow).

Gradual absorption of subretinal fluid was observed over 12 months of follow-up, and the BCVA improved to 20/30. The postoperative OCT examination confirmed the optic pit coverage by the inverted and folded ILM, as shown in figure 3.

## DISCUSSION

ODP is a rare and typically unilateral congenital cavitary anomaly of the optic disc. It is a herniation of a dysplastic retina into a collagen-rich excavation that often extends into the subarachnoid space through a lamina cribrosa defect. ODPs have been suggested to occur due to incomplete closure of the optic fissure during development, because of anomalous differentiation in the primitive epithelial papilla that allows abnormal microcommunication between the subarachnoid space surrounding the nerve and the pit^(1)^.

Although uncomplicated ODP remains asymptomatic, an ODP complicated with maculopathy (ODP-M) can cause severe visual impairment. These macular changes consist of serous detachment, cystic degeneration, and degenerative pigmentary changes. Its pathogenesis remains unclear. The accumulated fluid is thought to originate from either liquefied vitreous, cerebrospinal fluid, blood vessels at the base of the pit, or from the choroid^(2)^.

Surgical management is usually performed at the time of diagnosis; however, no established surgical technique exists. Laser photocoagulation alone to the temporal margin of the disc does not generally yield promising results. Possible side effects of laser photocoagulation near the disc include paracentral scotomas, no visual acuity changes, and low success rate in the resolution of SMD^(4)^.

Other proposed therapeutic modality include intravitreal gas injection alone, with the reasoning that pneumatic displacement will cause macular reattachment and VA improvement. This technique was used in a small series and resulted in visual improvement, although retinal reattachment was only achieved in about half of cases^(5)^.

An alternative approach proposed for the treatment of ODP-M is macular buckling surgery, fixing an implant to the posterior aspect of the globe along the 6-to-12 o’clock meridian. This procedure has been reported to achieve fluid resolution; however, its technique has not gained popularity since its introduction^(6)^.

The most widely accepted treatment for ODP maculopathy is a surgical approach involving PPV with or without ILM peeling, laser photocoagulation at the tem poral margin of the optic nerve head, and gas endotamponade^(7)^. ILM has been suggested as an important component of ODP maculopathy. Tangential and anteroposterior tractions are believed to facilitate the passage of fluid from the optic pit into the macula, and ILM peeling eliminates this tangential traction^(8)^.

Recently, several investigators have performed new procedures to directly seal the pit. The first description was of a case successfully treated with autologous platelet injection over the ODP^(9)^. Other techniques designed to seal the ODP using an autologous scleral flap and tissue fibrin sealant^(2)^.

Mohammed and Pai^(3)^ reported a case of pit covered with an autologous ILM flap, which led to the anatomical occlusion of the pit. Autologous tissue such the patient’s ILM can more physiologically seal the congenital defect in the lamina cribrosa, creating a permanent barrier and thus preventing the fluid flow through the pit. Good surgical outcomes in terms of restoration of macular anatomy and visual improvement were observed by the authors. Similar results were also obser ved in our case.

Obtaining the ILM as a single sheet with its pedicle still attached can be quite challenging technically; however, staining the ILM with brilliant blue dye helps create a large ILM flap. Postoperative ILM displacement from the optic disc with the pit is possible. However, leaving an area where the peeled ILM is still attached with a pedicle (or the stalk) and postoperative face-down positioning ensures that the ILM remains opposed to the new location^(3)^.

In conclusion, covering the pit with an inverted ILM flap is a reliable method to block the fluid flow from the ODP. This technique resulted in a satisfactory anatomic outcome with good functional improvement in BCVA. Further cases are needed to evaluate this technique.

## References

[1] Georgalas I, Ladas I, Georgopoulos G, Petrou P. (2011). Optic disc pit: a review. Graefe’s Arch Clin Exp Ophthalmol.

[2] Moisseiev E, Moisseiev J, Loewenstein A. (2015). Optic disc pit maculopathy: when and how to treat? A review of
the pathogenesis and treatment options. Int J Retin Vitreous.

[3] Mohammed OA, Pai A. (2013). Inverted autologous internal limiting membrane for management of
optic disc pit with macular detachment. Middle East Afr J Ophthalmol.

[4] Diab F, Al-sabah K, Al-Mujaini A. (2010). Successful surgical management of optic disc pit. maculopathy
without internal membrane peeling. Middle East Afr J Ophthalmol.

[5] Lincoff H, Kreissig I. (1998). Optical coherence tomography of pneumatic displacement of optic
disc pit maculopathy. Br J Ophthalmol.

[6] Theodossiadis GP, Theodossiadis PG. (2000). The macular buckling technique in the treatment of optic disk pit
maculopathy. Semin Ophthalmol.

[7] Hirakata A, Inoue M, Hiraoka T, McCuen BW. (2012). Vitrectomy without laser treatment or gas tamponade for macular
detachment associated with an optic disc pit. Ophthalmology.

[8] Shukla D, Kalliath J, Tandon M, Vijayakumar B. (2012). Vitrectomy for optic disk pit with macular schisis and outer
retinal dehiscence. Retina.

[9] Rosenthal G, Bartz-Schmidt KU, Walter P, Heimann K. (1998). Autologous platelet treatment for optic disc pit associated with
persistent macular detachment. Graefes Arch Clin Exp Ophthalmol.

